# The impact of blood MCP-1 levels on Alzheimer’s disease with genetic variation of UNC5C and NAV3 loci

**DOI:** 10.21203/rs.3.rs-3376348/v1

**Published:** 2023-09-28

**Authors:** Jinghan Huang, Yixuan Wang, Thor D. Stein, Ting Fang Alvin Ang, Yibo Zhu, Qiushan Tao, Kathryn L. Lunetta, Jesse Mez, Rhoda Au, Lindsay A. Farrer, Wei Qiao Qiu, Xiaoling Zhang

**Affiliations:** Boston University Chobanian & Avedisian School of Medicine; Boston University Chobanian & Avedisian School of Medicine; Boston University Chobanian & Avedisian School of Medicine; Boston University Chobanian & Avedisian School of Medicine; Boston University Chobanian & Avedisian School of Medicine; Boston University Chobanian & Avedisian School of Medicine; Boston University School of Public Health; Boston University Chobanian & Avedisian School of Medicine; Boston University Chobanian & Avedisian School of Medicine; Boston University Chobanian & Avedisian School of Medicine; Boston University Chobanian & Avedisian School of Medicine; Boston University Chobanian & Avedisian School of Medicine

**Keywords:** Alzheimer’s disease, blood, peripheral inflammation, MCP-1, genome-wide association study

## Abstract

**Background:**

Previous study shows that monocyte chemoattractant protein-1 (MCP-1), which is implicated in the peripheral proinflammatory cascade and blood-brain barrier (BBB) disruption, modulates the genetic risks of AD in established AD loci.

**Methods:**

In this study, we hypothesized that blood MCP-1 impacts the AD risk of genetic variants beyond known AD loci. We thus performed a genome-wide association study (GWAS) using the logistic regression via generalized estimating equations (GEE) and the Cox proportional-hazards models to examine the interactive effects between single nucleotide polymorphisms (SNPs) and blood MCP-1 level on AD in three cohorts: the Framingham Heart Study (FHS), Alzheimer’s Disease Neuroimaging Initiative (ADNI) and Religious Orders Study/Memory and Aging Project (ROSMAP).

**Results:**

We identified SNPs in two genes, neuron navigator 3 (*NAV3*, also named Unc-53 Homolog 3, rs696468) (p < 7.55×10^− 9^) and Unc-5 Netrin Receptor C (*UNC5C* rs72659964) (p < 1.07×10^− 8^) that showed an association between increasing levels of blood MCP-1 and AD. Elevating blood MCP-1 concentrations increased AD risk and AD pathology in genotypes of *NAV3* (rs696468-CC) and *UNC5C* (rs72659964-AT + TT), but did not influence the other counterpart genotypes of these variants.

**Conclusions:**

*NAV3* and *UNC5C* are homologs and may increase AD risk through dysregulating the functions of neurite outgrowth and guidance. Overall, the association of risk alleles of *NAV3* and *UNC5C* with AD is enhanced by peripheral MCP-1 level, suggesting that lowering the level of blood MCP-1 may reduce the risk of developing AD for people with these genotypes.

## Introduction

The relationship between peripheral proinflammatory factors and late-onset Alzheimer’s disease (AD) is largely unclear, and existing studies suggest that most of these factors have no or modest sensitivity and specificity for the prediction of AD risk.(1) Due to the blood-brain barrier (BBB), the effects of blood inflammatory factors on the brain are heavily regulated and vary between factors. Furthermore, inflammatory factors may interact with inherited genetic factors to modulate the risk for AD. For example, using the Framingham Heart Study (FHS), we found that within carriers of the AD risk genotype, apolipoprotein E4 (*APOE* 4), elevated blood C-reactive protein (CRP) levels are associated with increased AD risk.(2) This phenomenon is not observed among *APOE* 2 and *APOE* 3 carriers. Additionally, in ApoE4 carriers, elevated CRP is associated with the AD biomarker phosphorylated Tau (p-Tau) in the cerebral spinal fluid (CSF).(3)

Peripheral chronic inflammation causes a cascade of cytokines and chemokines to surge. Another CRP-related proinflammatory factor is monocyte chemoattractant protein-1 (MCP-1), which is also known as C-C motif chemokine ligand 2 (CCL2), is expressed in the blood and brain, and is implicated in inflammatory cell recruitment and BBB disruption.(4) MCP-1 is produced by macrophages and activated astrocytes during inflammation within the central nervous system. The expression of MCP-1 is also enhanced by CRP during chronic inflammation.(5, 6) The main function of MCP-1 as a chemoattractant is to drive leukocyte migration, especially monocytes/macrophages into damaged or infected tissues including AD brains(7–9), indicating that MCP-1 may play a role in activating microglia in the brain and thus leading to cognitive decline.(10–16) As reported, MCP-1 level in human CSF is associated with a faster rate of cognitive decline during the early stages of AD(13), and its overexpression in the brain promotes glial activation and accelerates tau pathology in a mouse model.(17) In addition, a higher plasma MCP-1 level is associated with greater severity of AD and mild cognitive impairment (MCI) and faster cognitive decline.(14) Recently, Cherry & Stein et al.(16) reported that MCP-1 protein levels in the dorsolateral prefrontal cortex (DLPFC) are correlated with the density of Iba1 + cells and CD68 + cells, increased chronic traumatic encephalopathy (CTE) severity, and are correlated with pTau independent of age at death and Aβ42 in AD and CTE.

However, the relationship between blood MCP-1 and the risk of developing AD is not consistent across different studies.(1) We hypothesized that the levels of blood MCP-1 differently impact vulnerable people with AD risk genes, to increase AD risk. Our previous study discovered that the blood MCP-1 level can modulate the genetic risks of AD for two established AD gene loci, *APOE* and *HLA-DRB1*.(18) However, there is no genome-wide search for the interaction effects between blood MCP-1 levels with genetic variants on AD risk. In this study, we conducted a Gene-by-Environment (G×E) genome-wide association study (GWAS) by applying logistic regression utilizing generalized estimating equations (GEE). Top findings were further tested with Cox proportional hazards models for the incidence of AD. By analyzing data from the FHS and the Alzheimer’s Disease Neuroimaging Initiative (ADNI), for the first time, at the genome-wide significance level (p < 5.0×10^− 8^) we found that elevated blood MCP-1 increased AD risk, hippocampal atrophy, and AD neuropathology only in people carrying one genotype of SNPs in two gene loci: Neuron navigator 3 (*NAV3* rs696468) and Unc-5 Netrin Receptor C (*UNC5C* rs72659964). Interestingly, both genes have been identified in *C. elegans* to be involved in neuron outgrowth and guidance and both are associated with AD in humans.(19, 20)

Further explorations in the ROSMAP cohort provided molecular mechanism evidence of the significant associations between AD and variant in *NAV3* at multi-omics levels (i.e., DNA methylation, gene expression).

## Methods

### Participants

#### Framingham Heart Study

**Figure 1:**
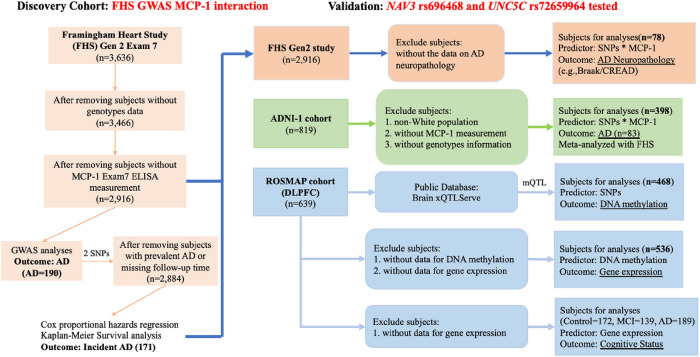
Flowchart for Sample Selection and Study Design

The Framingham Heart Study (FHS) is a single-site, multi-generation, community-based, prospective cohort study in Framingham, Massachusetts.(2) Surveillance for incident AD/dementia was initiated in 1975 and diagnoses of AD and other dementia causes are adjudicated through consensus panel.(21) This study focused on the offspring cohort (Generation 2) participants who have data on genome-wide genotyping and serum MCP-1 measurement from Exam 7 (baseline) and followed up on AD/dementia outcomes up to 2019 (Fig. 1). Informed consent was obtained from all study participants, and the study protocol was approved by the Institutional Review Board of Boston University Medical Campus.

#### Alzheimer’s Disease Neuroimaging Initiative (ADNI)

To validate the findings in the FHS, we used the data from ADNI-1, which is a longitudinal multicenter study that was launched in 2003 as a public-private partnership. It was designed to test whether neuroimaging, biological markers in CSF and blood, and clinical and neuropsychological assessments can be combined to predict the diagnosis and progression of AD.(22) Participants underwent longitudinal in-depth neuropsychological evaluations(23) and consensus diagnoses of cognitive normal (CN), mild cognitive impairment (MCI), and AD were assigned based on established research diagnostic criteria(24). Participants with blood MCP-1 measured were included in this analysis. Baseline blood MCP-1 was used. After filtering out self-reported non-White subjects and those without MCP-1 measurement and genotype information, 398 ADNI-1 participants were included in the analysis (Fig. 1).

#### Religious Orders Study/Memory and Aging Project (ROSMAP)

ROSMAP data was further used to investigate whether *cis*-regulatory variants affect DNA methylation and gene expression in brain and their relationships with AD (Fig. 1). The ROS is a longitudinal, epidemiologic clinical-pathological study of memory, motor, and functional problems in older Catholic nuns, priests, and brothers aged 65 years and older from across the United States. Since 1994, approximately 1,200 older persons have been enrolled. The MAP is a longitudinal, epidemiologic clinical-pathologic study of dementia and other chronic diseases of aging. Older persons are recruited from about 40 continuous care retirement communities and senior subsidized housing facilities around the Chicago metropolitan area. MAP began in 1997 and over 1,600 older adults have enrolled. ROSMAP participants have yearly blood draws which result in the storage of serum, plasma, and cells. Clinical evaluation, self-report, and medication inspection are used to document medical conditions. Subjects are evaluated neurologically every year, and a review of all antemortem data at the time of death leads to a final clinical diagnosis for each participant: each individual receives a diagnosis of syndromic Alzheimer’s disease (AD), of mild cognitive impairment (MCI), or of no cognitive impairment (NCI). Details have been previously described.(25)

#### Plasma MCP-1 measurement

MCP-1 levels (Exam 7) in FHS participants were measured using enzyme-linked immunosorbent assay (ELISA) with a Dade Behring BN100 nephelometer(26) from fasting blood samples that were collected at exam 7 from the antecubital vein (details had been previously described).(27) In ADNI-1, MCP-1 in plasma samples was collected using the Human Discovery MAP Panel and measurement platform.(28, 29) MCP-1 level was log-transformed in the downstream analysis as a continuous variable. The median of the MCP-1 level was also used to define participants into low versus high MCP-1 groups as a categorical variable.

#### AD neuropathology

A subset of the FHS Offspring participants (n = 105) donated their brains, which were used for neuropathological characterization. For data analysis, three routine neuropathology variables were selected, including the Braak stage for neurofibrillary degeneration, the CERAD score for density of neocortical neuritic plaques, and the CERAD semi-quantitative score for diffuse plaques. Neuropathological brain evaluation was performed by neuropathologists blinded to all demographic and clinical information.(30) 78 participants had the above neuropathology variables and blood MCP-1 measurement were included in the analysis (**Figure S1**).

#### Genome-wide association studies in the FHS

The genome-wide association studies (GWAS) were performed using the Framingham analytical pipeline for common autosomal single nucleotide polymorphisms (SNPs) imputed using MACH with the 1000G European Ancestry reference panel from March 2012. In brief, a total number of 412,053 genotyped SNPs were used as input to the MACH program (http://genome.sph.umich.edu/wiki/minimac) for phasing. The final genotype set was obtained by following the GIANT protocol for imputation(31) to the November 2010 release of 1000G EUR panel based only on individuals of European descent. The relationship between AD and the interaction term of MCP-1 and SNP dosage was tested using GEEPACK (logistic regression utilizing generalized estimating equations; accounts for relatedness in FHS) while adjusting for age, sex, years of education, and the first 10 principal components (PCs). Additive models were assumed. Only autosomal SNPs (chr1–22) with minor allele frequency (MAF) ≥ 5% were chosen for the analysis to reduce false positives due to the sample size of FHS. Additionally, samples with genotyping rate less than 97% and with an excess number of heterozygote observations (P < 10^− 6^) or Mendelian errors were removed. The Manhattan plot, QQ plot and genomic control(32) were used for visualization quality control and accounting for genomic inflation. LocusZoom(33) was used to present the regional information. P values < 5.0×10^− 8^ were considered genome-wide statistically significant for the SNP-MCP-1 interactive effects for AD.

#### DNA methylation and Gene expression analysis

ROSMAP data was used to investigate whether cis-regulatory variants (SNPs within 5KB for mQTL and 1 MB for eQTL of neighboring genes) affect DNA methylation (mQTL) and gene expression (eQTL) in brain as well as their relationships with AD. mQTL summary results for selected variants were obtained from Brain xQTLServe for ROSMAP (http://mostafavilab.stat.ubc.ca/xQTLServe/). Genotype data were generated from 2,093 individuals of European descent. Of these individuals, DNA methylation array (the Illumina Infinium HumanMethylation 450K BeadChip) data was derived from fresh frozen post-mortem brain samples collected from the dorsolateral prefrontal cortex (DLPFC) region of 468 subjects. Gene expression RNA-seq (Illumina HiSeq) data were generated from the DLPFC of 494 individuals with an average sequencing depth of 90 million reads. A detailed description was previously described.(34) Furthermore, we examined the gene expression patterns across different AD brain regions using the Agora database (https://agora.adknowledgeportal.org/genes), which includes Cerebellum (CBE), DLPFC, Frontal Pole (FP), Inferior Frontal Gyrus (IFG), Parahippocampal Gyrus (PHG), Superior Temporal Gyrus (STG) and Temporal Cortex (TCX). This database was initially developed by the NIA-funded AMP-AD consortium that shared evidence in support of AD target discovery.

#### Statistical analysis

Analyses were performed using the R statistical environment (R 3.6.2). Several variables including sample size, age at baseline, sex, years of education, *APOE* ε4 status, and incident AD status were summarized as the baseline characteristics stratified by MCP-1 levels. Group differences were assessed by analysis of variance (ANOVA) for normally distributed continuous variables, by Kruskal–Wallis rank sum test for continuous variables with skewed distributions, and by Chi-Square test of independence for categorical variables.

In stratified genotype analysis, logistic regression was performed for AD status (prevalent + incident AD), using MCP-1 level (log-transformed continuous or high vs. low group) as the predictor, and adjusting for covariates (age at MCP-1 measurement, sex, years of education, and the first 5 PCs). Additionally, both Cox proportional hazards regression and Kaplan-Meier survival analysis were conducted for AD incidence. Heterozygous and homozygous minor allele genotypes were combined in this section due to the relatively low MAF of the two SNPs (0.14 for rs696468 and 0.06 for rs72659964).

For neuropathology, ordinal regression was performed to examine whether the interactions between SNPs and MCP-1 associate with AD neuropathology traits including Braak stage, diffuse plaque CERAD score, neuritic plaque CERAD score, adjusting for the same covariates as described above.

To support the findings in the FHS, the ADNI-1 cohort was analyzed using logistic regression models adjusted for age at baseline, sex, and years of education, then the results were meta-analyzed with that of FHS by inverse-variance weighted meta-analysis using METAL.(35)

## Results

### Characteristics of the FHS population

The 2,884 FHS Generation 2 participants included in this study (Table 1) had an average follow-up period of 18.5 years (from exam 7), and 171 (5.93%) of the participants developed AD during this follow-up. Subjects were divided into four quartiles based on the MCP-1 serum concentration measured at baseline (Exam 7). Participants with the lowest MCP-1 concentration (first quantile) were the youngest (p < 0.001) and had the highest years of education (p < 0.001), compared to participants with higher MCP-1 quartiles. Sex and *APOE* 4 genotypes did not show significant differences across MCP-1 quartiles. Although there was an overall increase in the incidence of AD with increasing concentration of blood MCP-1 quartile, the relationship was not linear (3.61% vs. 6.38% vs. 4.72% vs. 9.02%, p < 0.001), (Table 1). In addition, when directly testing the associations between continuous MCP-1 concentration and AD incidence after adjusting for confounders including age, sex and years of education, no statistically significant relationship was found (data not shown). Since we found that MCP-1 impacts the AD risk of genetic variants in APOE and *HLA-DRB1* differently(18), we hypothesized that blood MCP-1 might modulate the effects of other genetic variants on AD risk.

### Interactions between GWAS-selected SNPs and blood MCP-1 on AD risk in the FHS

**Figure 2:**
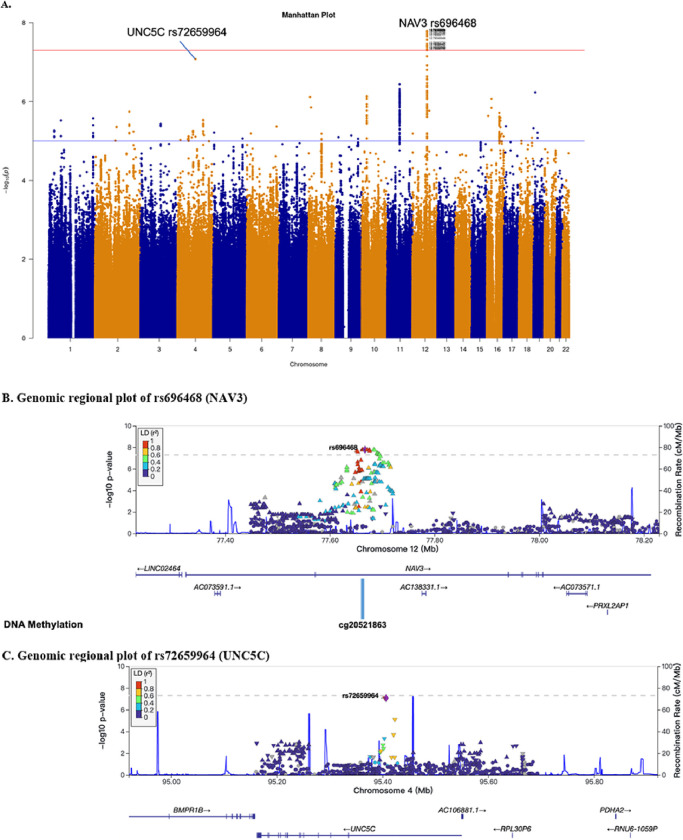
Manhattan plot and Genomic regional plots for the G×E GWAS analyses between MCP-1 and genomic variants

To genome-wide search genetic loci interacting with peripheral blood MCP-1 level to affect AD risk beyond established AD loci, we conducted a G×E GWAS for AD with an interaction term between MCP-1 and SNP as predictor using a logistic regression model (Fig. 2 and **Figure S1**). As shown in the Manhattan plot after genomic control (Fig. 2A), 20 SNPs showed suggestive significance (p < 1.0×10^− 7^). One locus on chromosome 12 showed genome-wide significant interactions with MCP-1 on AD risk (p < 5.0×10^− 8^) (**Table S1**), including 18 SNPs in intronic region of gene *NAV3* (rs696468 (MAF = 0.14) as sentinel SNP) as shown in Fig. 2B. Another locus on chromosome 4 reached suggestive significance (p < 1.0×10^− 7^) including two SNPs in intronic region of *UNC5C* (rs72659964 (MAF = 0.06) as sentinel SNP), as presented in Fig. 2C.

**Figure 3:**
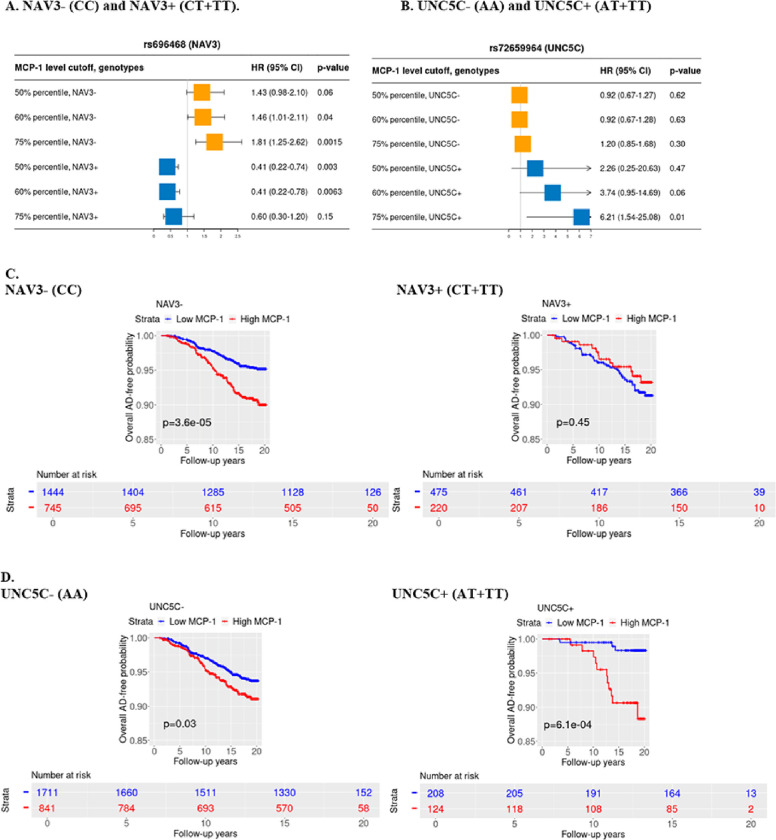
Kaplan-Meier survival plot and Forest plot of the stratified genotype analysis for the effect of MCP-1 levels on incident AD in Cox proportional hazard regression models

Among SNPs passing a genome-wide significance (p < 5.0×10^− 8^), sentinel SNP rs696468 in *NAV3* was selected for further analyses. Another significant SNP rs72659964 (p < 1.0×10^− 7^ in FHS and p < 5.0×10^− 8^ in meta-analysis) in *UNC5C* was also selected for further analyses since *UNC5C* is a homolog of *NAV3* and is associated with familial and sporadic AD.(36–38) As shown in Table 2, the SNP main effect alone on AD risk was not significant (p > 0.05). However, their interaction effects with the continuous MCP-1 level (log-transformed) on AD is significant (p = 1.64×10^− 8^ for *NAV3* and p = 8.36×10^− 8^ for *UNC5C* in FHS) using logistic regression models. We next performed SNP stratified analysis to test the association between MCP-1 and AD incidence using Cox proportional hazards regression models for AD risk after adjusting for age at baseline, sex, years of education, and PCs. Due to the relatively low MAF of the two SNPs (0.14 for rs696468 and 0.06 for rs72659964), heterozygous and homozygous minor allele genotypes were combined. As shown in **Table S2–3**, elevated MCP-1 concentration (log-transformed) was associated with a higher incidence of AD among *NAV3* rs696468-CC carriers (HR = 2.68, 95% CI = 1.55, 4.62, p = 3.9×10^− 4^). On the other hand, the elevated MCP-1 was negatively associated with AD risk among *NAV3* rs696468-CT + TT carriers (HR = 0.25, 95% CI = 0.10, 0.60, p = 0.002). These dose-dependent relationships were also observed using different MCP-1 percentile cutoffs (Fig. 3A). Elevated MCP-1 concentration (log-transformed and percentile cutoffs) was associated with a higher incidence of AD among *UNC5C* rs72659964-AT + TT carriers (HR = 80.74, 95% CI = 8.32, 783.28, p = 1.5×10^− 4^), but this increasing trend was largely attenuated for the *UNC5C* rs72659964-AA carriers (**Table S2** and Fig. 3B). In Kaplan-Meier analyses, we further stratified subjects into low and high MCP-1 group, and significantly lower AD-free probability was observed among subjects having high blood MCP-1 (75% percentile as cutoffs) and among subjects with *NAV3* rs696468-CC carriers (p = 3.6×10^− 5^, Fig. 3C) and subjects with *UNC5C* rs72659964-AT + TT carriers (p = 6.1×10^− 4^, Fig. 3D) (50% percentile as cutoffs in **Figure S2**). In contrast, elevated MCP-1 showed no or attenuated positive associations with AD risk among persons with other genotypes for these SNPs.

### Validation of the interactive effects of blood MCP-1 and SNPs in NAV3 and UNC5C for AD in the ADNI cohort

To replicate the findings in the FHS, we analyzed the ADNI-1 cohort in the same way as for the FHS using logistic regression models and adjusted for the same confounders. We then meta-analyzed the summary statistic results from the FHS and ADNI-1 with the inverse-variance weighted method for the 21 suggestive significant SNPs (Table 2, Table 3 and **Table S4**). Indeed, this analysis increased the significance as we obtained genome-wide significant interactions for all the selected SNPs (P < 5.0×10^− 8^) (Table 2 and **Table S4**). In genotype stratification analysis, we observed the same direction effects of elevated blood MCP-1 for increasing AD risk in rs696468-CC (Z = 3.94, p = 8.1×10^− 4^) and *UNC5C* rs72659964-AT + TT (Z = 3.35, p = 8.0×10^− 4^) carriers in both cohorts (Table 3), while in other genotypes the associations were inconsistent or insignificant between the two cohorts.

### Associations of blood MCP-1 and brain neuropathology among different genotypes

To further investigate the impact of peripheral MCP-1 on AD brain pathology, the relationships between blood MCP-1, selected variants, and three neuropathology features were evaluated using ordinal regression in the FHS, in which 78 participants donated their brain after death. As shown in Table 4, consistently, *NAV3* rs696468 significantly interacted with blood MCP-1 concentration for Braak score (p = 0.02), CERAD score (neocortical neuritic plaque) (p = 0.03), and CERAD semi-quantitative score (diffuse plaques) (p = 0.03). *UNC5C* rs72659964 significantly interacted with blood MCP-1 concentration for CERAD semi-quantitative score (diffuse plaques) (p < 0.001). The main effects of the two SNPs without counting peripheral MCP-1 for AD pathology were not significant.

### Exploration of regulatory functions of variants in NAV3 and UNC5C underlying AD

**Figure 4:**
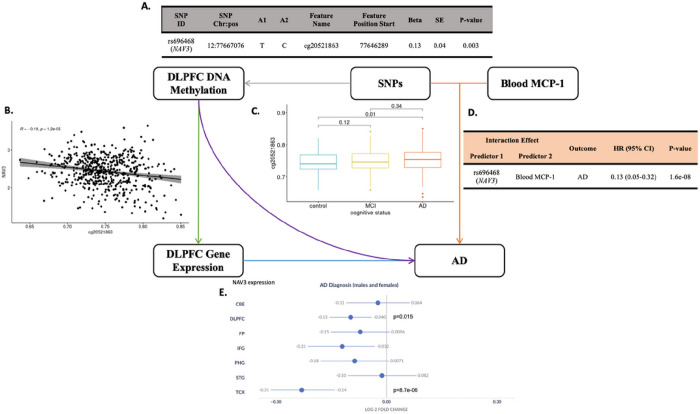
The relationships between SNPs, Blood MCP-1, DLPFC DNA methylation, DLPFC gene expression and AD

To investigate possible regulatory mechanism of rs696468 in *NAV3*, and whether *NAV3* is the function target gene of rs696468, we first performed mQTL and eQTL mapping by leveraging the available genotype, and DNA methylation and RNA-seq gene expression measured from the same DLPFC brain region in ROSMAP. (Fig. 4). Interestingly, *NAV3* rs696468 was negatively associated with methylation level of CpG site cg20521863 (located within *NAV3*) (p = 0.003) (Fig. 4A), and the cg20521863 methylation level was negatively associated with *NAV3* gene expression (p = 1.2×10^− 5^) (Fig. 4B). Of note, AD had a higher methylation level of cg20521863 (p = 0.01) compared with normal controls in DLPFC region of ROSMAP participants (Fig. 4C). Consistently, using the Agora database, we discovered that NAV3 had a significantly lower level of expression in DLPFC (p = 0.02) and temporal cortex region (TCX) (p = 8.7×10^− 6^) among AD cases compared with normal controls (Fig. 4E). Both *NAV3* and *UNC5C* showed relatively high expression levels in brain related tissues in the Genotype-Tissue Expression (GTEx) database (**Figure S3**).

Meanwhile, we did not observe significant associations for *UNC5C* rs72659961/rs72659964 in any molecular QTL mapping databases we studied (data not shown). The regulatory functions of these variants should be further investigated in the future.

## Discussion

Our study conducted G×E GWAS analysis to investigate the interactive influence of genetic and internal environmental factors on AD development. Through this analysis, we discovered two genetic loci, *NAV3* rs696468 and *UNC5C* rs72659964, that are implicated in AD under the influence by blood MCP-1 levels (Fig. 2, Table 2). Specifically, we found that elevated blood MCP-1 levels were associated with a higher risk of AD, but only among individuals who carry specific alleles of the *NAV3*-rs696468-CC and *UNC5C*-rs72659964-AT + TT (Fig. 3, **Table S2, and** Table 3). In contrast, individuals with different genotypes of these genetic variants did not exhibit the same association between MCP-1 levels and AD risk. Additionally, our study revealed a correlation between increased blood MCP-1 concentrations and older age (Table 1). This finding aligns with previous research, indicating that advancing age is linked to peripheral chronic inflammation leading to neurodegeneration.(39) Consequently, our study proposes a novel mechanism whereby proinflammatory factors in the peripheral system in aging may heighten the risk of neurodegenerative changes in the brain.

*NAV3*, also named Unc-53 Homolog 3, is a homolog of *UNC5C* which has a similar cellular function as *UNC5C* in the nervous system in C. elegans.(40) It is reported that some microRNAs, e.g., miR-29c, regulates *NAV3* protein expression in an AD mouse model.(41) In a recent study, the association of AD and *NAV3* was further highlighted by an AI-based approach using data from ROSMAP and Mayo RNAseq Study (https://adknowledgeportal.org).(42) Our further explorations in the ROSMAP cohort at epigenomic and transcriptomic levels (Fig. 4) indicate that *NAV3* rs696468 plays a role in DNA methylation, which, in turn, is associated with *NAV3* expression. Interestingly, we observed negative associations between *NAV3* expression and AD in DLPFC and temporal cortex. It is possible that blood MCP-1 levels may act as a moderator for the association between AD and *NAV3* expression, which is shown to be impacted by the *NAV3* genetic loci.

*NAV3* and *UNC5C* are homologs and have been identified in C. elegans to have neuron outgrowth and guidance and are associated with AD in humans.(19, 20, 43, 44) Mechanistic studies on cell and animal models discovered that aberrant *UNC5C* might contribute to AD by activating death-associated protein kinase 1 (DAPK1) which is involved in AD pathogenesis with extensive involvement in aberrant tau, Aβ and neuronal apoptosis/autophagy.(20, 45) In addition, deleting *UNC5C* from netrin-1–depleted mice can mitigate AD pathologies and reduces cognitive disorders. The δ-secretase truncates UNC5C and increases its neurotoxicity, contributing to AD pathogenesis.(36) In brain imaging, *UNC5C* loci has been reported to be associated with temporal volume and alter the atrophy of strategic regions of AD such as the hippocampus and precuneus.(46)

This study has several limitations. First, the FHS cohort did not undergo lumbar puncture (LP) to obtain CSF levels of MCP-1. Second, due to the small sample size and age difference in the ADNI cohort, we only observe the interactive genotype-MCP-1 effects on AD in the same direction as in the FHS, but not statically significant. Since the ADNI study lacks information on the time of AD onset, time-to-event analysis cannot be performed. Third, the number of samples with brain neuropathology was small. The two SNPs have relatively low MAF (0.14 for *NAV3* rs696468 and 0.06 for *UNC5C* rs72659964), limiting the genotype stratification analysis for AD neuropathology (n = 78) which results in a wide 95% CI. In addition, the functions for *UNC5C* loci tagged by rs72659964 at epigenomic/transcriptomic levels remain unclear in the current datasets we analyzed, therefore further investigation should be performed for this locus using different/larger omics datasets.

## Conclusions

Our study suggests a novel mechanism that proinflammatory factors in the peripheral system in aging may heighten the risk of neurodegenerative changes in the brain. By applying the Gene-by-Environment GWAS and using three cohorts to assess clinical outcomes, AD neuropathology and genetic functions at multi-omics levels, our study reveals an interactive effect of increased blood MCP-1 levels on AD risk within two AD novel loci. This finding emphasizes the importance of considering individual genotypes for precision medicine approaches when assessing the impact of specific peripheral inflammatory factors on AD risk. Future studies are needed to explore the effect of blood MCP-1 levels on AD and AD-related endophenotypes in larger postmortem cohorts, especially those with multiethnic representation, to assess the generalizability of our findings. As multiple MCP-1 inhibitors are currently being developed and tested in clinical trials for cancer and autoimmune diseases(47), these medications could be repurposed for AD intervention and prevention in allele carriers of *NAV3* rs696468-CC and *UNC5C* rs72659964-AT + TT with peripheral inflammation.

## Figures and Tables

**Table 1:** Basic characteristics, APOE, CRP and incidence of AD among different MCP-1 level groups in FHS

Characteristics	all subjects	First quartile (31.1, 253.1), pg/ml	Second quartile (253.1, 312.3), pg/ml	Third quartile (312.3, 382.9), pg/ml	Fourth quartile (382.9, 2139.8), pg/ml	P-value^d^
**N subjects, No. (%)**	2884	721 (25)	721 (25)	721 (25)	721 (25)	NA
**Incidence of AD, No. (%)**	171 (5.93)	26 (3.61)	46 (6.38)	34 (4.72)	65 (9.02)	<0.001^c^
**Age when measuring MCP-1, mean (SD)**	60.64 (9.27)	57.65 (9.22)	60.57 (9.12)	61.52 (8.75)	62.83 (9.22)	<0.001^a^
**Follow-up years, median (Q1-Q3)**	18.46 (14.87–19.41)	18.78 (17.31–19.60)	18.50 (15.07–19.44)	18.39 (15.00–19.26)	17.93 (12.52–19.22)	<0.001^b^
**Male, No. (%)**	1332 (46.19)	316 (43.83)	323 (44.80)	349 (48.40)	344 (47.71)	0.24^c^
**Years of education, mean (SD)**	13.99 (2.44)	14.31 (2.41)	14.18 (2.56)	13.89 (2.33)	13.57 (2.40)	<0.001^a^
**APOEε4**^e^, **No. (%)**	573 (20.21)	126 (17.48)	148 (20.53)	144 (19.97)	155 (21.50)	0.31^c^

A total of 2,884 subjects in the FHS were divided into four quartiles based on blood MCP-1 levels in the analysis. Means (SD) and Medians (Q1 = Q3) were reported. ANOVA was used to analyze continuous variables, while n (%) with the χ^2^ test was used for categorical variables for the MCP-1 quartile comparisons. P values indicating statistical significance are shown.

Abbreviations: AD, Alzheimer’s disease; MCP-1, Monocyte Chemoattractant Protein-1.

a. ANOVA test p-value.

b. Rank Sum test p-value.

c. χ^2^ test p-value.

d. P-value of comparison between four groups

e. *APOE* ε4 = 34 + 44

**Table 2:** The relationships between AD, variants and the interaction of both genotypes and MCP-1 using logistic regression

Effect/Gene	NAV3 rs696468		UNC5C rs72659964	
	OR (95%CI)	P-value	OR (95%CI)	P-value
**FHS**				
SNP main effect^a^	1.34 (1.00–1.79)	0.05	0.73 (0.45–1.20)	0.21
Interaction with MCP-1^b^	0.08 (0.04–0.19)	**1.6×10^− 8^**	145.5 (25.4–832.5)	**8.4×10^− 8^**
**ADNI**				
Interaction with MCP-1	0.65 (0.04–11.38.)	0.77	27.11 (0.11–6952.11)	0.24
**Meta-analysis (FHS + ADNI)** ^c^	**Z-score**	**P-value**	**Z-score**	**P-value**
Interaction with MCP-1	−5.78	**7.6×10^− 9^**	5.72	**1.07×10^− 8^**

Using the FHS&ADNI datasets and logistic regression model, relationships and interactive effects between continuous blood MCP-1 levels and *NAV3* rs696468 and *UNC5C* rs72659964 on AD risk were examined and shown.

a. AD ~ SNP dosage + age + sex + years of education + PCs

b. AD ~ SNP dosage:MCP-1 (log transformed) + SNP dosage + MCP-1 + age + sex + years of education + PCs; GWAS results using GEEPACK.

c. Fixed effect model was applied.

**Table 3:** Stratified genotype analysis of MCP-1 levels on AD, adjusted by age, sex, years of education using logistic regression

Genes	Genotypes	FHS		ADNI^a^		FHS + ADNI meta-analysis^b^		
		Beta (SE)	P-value	Beta (SE)	P-value	Z-score	P-value	Direction (FHS + ADNI)
**NAV3 rs696468**	CC	1.04 (0.27)	**1.4×10^−4^**	0.67 (0.58)	0.25	3.94	**8.1×10^−5^**	++
	CT + TT	−1.56 (0.49)	**0.001**	0.33 (1.45)	0.82	−2.96	**0.003**	−+
**UNC5C rs72659964**	AA	0.22 (0.24)	0.36	0.49 (0.56)	0.39	1.19	0.23	++
	AT + TT	3.17 (1.06)	**0.003**	6.18 (3.61)	0.09	3.35	**8.0×10^−4^**	++

FHS and ADNI-1 participants were divided into subgroups based on the genotype of *NAV3* rs696468 CC vs. CT + TT or the genotype of *UNC5C* rs72659964 AA vs. AT + TT, and an analysis of the effects of blood MCP-1 levels (log-transformed) on AD was performed after stratification by genotype. Logistic regression model was used with adjustments for age, sex and years of education. Heterozygous and homozygous minor allele genotypes were combined due to the relatively low MAF of the two SNPs (0.14 for rs696468 and 0.06 for rs72659964).

a. Baseline blood MCP-1 was used in ADNI-1.

b. AD ~ log (MCP-1) + age + sex + years of education, stratified by different genotypes with fixed-effect model.

**Table 4:** Relationship between Neuropathology scores and NAV3 or UNC5C alone vs. the interaction of either genotype with MCP-1 concentration using ordinal regression

Neuropathology Effect/Gene	NAV3 rs696468		UNC5C rs72659964	
Braak stage	estimate (SE)	P-value	estimate (SE)	P-value
SNP effect^a^	0.71 (0.43)	0.09	0.17 (0.65)	0.80
Interaction with MCP-1^b^	−3.81 (1.66)	**0.02**	2.40 (3.73)	0.52
**CERAD score (neuritic plaque)**	**estimate (SE)**	**P-value**	**estimate (SE)**	**P-value**
SNP effect	0.59 (0.43)	0.17	−0.47 (0.64)	0.46
Interaction with MCP-1	−3.57 (1.61)	**0.03**	6.84 (8.81)	0.44
**CERAD score (diffuse plaques)**	**estimate (SE)**	**P-value**	**estimate (SE)**	**P-value**
SNP effect	0.94 (0.43)	**0.03**	−0.09 (0.73)	0.90
Interaction with MCP-1	−3.34 (1.57)	**0.03**	10.09 (0.19)	**< 0.001**

Using the FHS dataset and ordinal regression model, relationships and interactive effects between continuous blood MCP-1 levels (log transformed) and *NAV3* rs696468 and *UNC5C* rs72659964 on AD neuropathology scores were examined and shown (n = 78).

a. SNP effect: AD neuropathology ~ SNPs + age + sex + years of education + PCs

b. Interaction: AD neuropathology ~ SNPs:log (MCP-1) + SNPs + log (MCP-1) + age + sex + years of education + PCs

## Data Availability

The FHS data is available at dbGaP (https://www.ncbi.nlm.nih.gov/projects/gap/cgi-bin/study.cgi? study_id=phs000007.v33.p14 ) upon reasonable request/application. The ADNI data is available at https://adni.loni.usc.edu/ upon reasonable request/application. The ROSMAP data is available at the AD Knowledge Portal (https://adknowledgeportal.org).

## Abbreviations

MCP-1: monocyte chemoattractant protein-1
BBB: blood-brain barrier
AD: Alzheimer’s disease
GWAS: genome-wide association study
GEE: generalized estimating equations
SNP: single nucleotide polymorphism
FHS: Framingham Heart Study
ADNI: Alzheimer’s Disease Neuroimaging Initiative
ROSMAP: Religious Orders Study/Memory and Aging Project
CCL2: C-C motif chemokine ligand 2
CRP: C-reactive protein
CSF: Cerebrospinal Fluid
MCI: mild cognitive impairment
DLPFC: dorsolateral prefrontal cortex
CTE: chronic traumatic encephalopathy
CN: cognitive normal
NCI: no cognitive impairment
ELISA: enzyme-linked immunosorbent assay
PC: principal component
MAF: minor allele frequency
mQTL: methylation quantitative trait locus
eQTL: expression quantitative trait locus
CBE: Cerebellum
FP: Frontal Pole
IFG: Inferior Frontal Gyrus
PHG: Parahippocampal Gyrus
STG: Superior Temporal Gyrus
TCX: Temporal Cortex
ANOVA: analysis of variance
LP: lumbar puncture
